# Quantitative Ultrashort Echo Time Magnetization Transfer Imaging of the Osteochondral Junction: An In Vivo Knee Osteoarthritis Study

**DOI:** 10.3390/jimaging11060198

**Published:** 2025-06-16

**Authors:** Dina Moazamian, Mahyar Daskareh, Jiyo S. Athertya, Arya A. Suprana, Saeed Jerban, Yajun Ma

**Affiliations:** 1 Department of Radiology, University of California, San Diego, CA 92037, USA; mdaskareh@health.ucsd.edu (M.D.); asuprana@ucsd.edu (A.A.S.); sjerban@health.ucsd.edu (S.J.); yam013@health.ucsd.edu (Y.M.); 2 Department of Bioengineering, University of California, San Diego, CA 92037, USA

**Keywords:** osteoarthritis (OA), osteochondral junction (OCJ), ultrashort echo time (UTE) MRI, macromolecular fraction (MMF), magnetization transfer ratio (MTR)

## Abstract

Osteoarthritis (OA) is the most prevalent degenerative joint disorder worldwide, causing significant declines in quality of life. The osteochondral junction (OCJ), a critical structural interface between deep cartilage and subchondral bone, plays an essential role in OA progression but is challenging to assess using conventional magnetic resonance imaging (MRI) due to its short T2 relaxation times. This study aimed to evaluate the utility of ultrashort echo time (UTE) MRI biomarkers, including macromolecular fraction (MMF), magnetization transfer ratio (MTR), and T2*, for in vivo quantification of OCJ changes in knee OA for the first time. Forty-five patients (mean age: 53.8 ± 17.0 years, 50% female) were imaged using 3D UTE-MRI sequences on a 3T clinical MRI scanner. Patients were stratified into two OA groups based on radiographic Kellgren–Lawrence (KL) scores: normal/subtle (KL = 0–1) (*n* = 21) and mild to moderate (KL = 2–3) (*n* = 24). Quantitative analysis revealed significantly lower MMF (15.8  ±  1.4% vs. 13.6 ± 1.2%, *p* < 0.001) and MTR (42.5 ± 2.5% vs. 38.2  ±  2.3%, *p* < 0.001) in the higher KL 2–3 group, alongside a higher trend in T2* values (19.7  ±  2.6 ms vs. 21.6  ±  3.8 ms, *p* = 0.06). Moreover, MMF and MTR were significantly negatively correlated with KL grades (r = −0.66 and −0.59; *p* < 0.001, respectively), while T2* showed a weaker positive correlation (r = 0.26, *p* = 0.08). Receiver operating characteristic (ROC) analysis demonstrated superior diagnostic accuracy for MMF (AUC = 0.88) and MTR (AUC = 0.86) compared to T2* (AUC = 0.64). These findings highlight UTE-MT techniques (i.e., MMF and MTR) as promising imaging tools for detecting OCJ degeneration in knee OA, with potential implications for earlier and more accurate diagnosis and disease monitoring.

## 1. Introduction

Osteoarthritis (OA) is the most prevalent degenerative joint disease worldwide, causing pain, impaired mobility, and reduced quality of life in millions of individuals [1,2]. The disease is characterized by progressive articular cartilage deterioration, subchondral bone remodeling, and synovial inflammation [3].

The osteochondral junction (OCJ) serves as a critical interface between deep cartilage and the subchondral bone [4,5], comprising unmineralized radial cartilage, the tidemark, mineralized cartilage, and the subchondral bone plate [6]. In addition to its role in mechanically supporting cartilage, the OCJ may also contribute to OA pathogenesis through processes such as vascularization [7,8], cracking [9], and ossification [7]. Therefore, detecting alterations at the OCJ could be important for diagnosing OA [10].

Magnetic resonance imaging (MRI) is a widely used imaging modality for detecting OA in the knee [11,12]. However, conventional MRI techniques, such as fast spin echo (FSE) and gradient echo (GRE), yield minimal or no signal from the OCJ due to its extremely short transverse relaxation times (short T2 or T2*) [13,14]. To overcome this limitation, ultrashort echo time (UTE) MRI sequences, characterized by echo times shorter than 100 μs, have been developed to effectively visualize regions with short T2 components in the OCJ [14,15].

To investigate the OCJ morphology, several UTE sequences have been developed, including dual-echo UTE [15,16], fat-saturated T1-weighted UTE [17], single inversion recovery (IR) fat-suppressed UTE [18], and dual inversion recovery (DIR) UTE techniques [19,20]. These techniques utilize long T2 signal suppression methods, such as echo subtraction or IR signal suppression, to highlight short T2 and T1 OCJ regions.

In addition to morphological UTE-MRI, quantitative UTE-based techniques, including UTE magnetization transfer (UTE-MT), UTE-T1ρ [21], and UTE-T2*, have been developed for assessing articular cartilage degeneration in OA [17,22,23,24,25,26]. Among these methods, the UTE-MT technique is insensitive to the magic angle effect and allows the quantification of multiple parameters, such as the macromolecular fraction (MMF), magnetization transfer ratio (MTR), macromolecular relaxation time (T2mm), and proton exchange rate in short T2 tissues [27,28]. MMF and MTR have demonstrated potential in detecting cartilage degeneration associated with OA [23,27]. Previous ex vivo studies have explored the feasibility of using MMF and T2* to quantitatively evaluate the collagen-rich OCJ [25]. However, comprehensive in vivo studies comparing UTE-MT biomarkers to other UTE-based metrics, like UTE-T2*, specifically for OCJ assessment in knee OA, remain limited.

This study aimed to evaluate the feasibility of quantitative UTE-MT biomarkers of MMF and MTR for detecting OCJ changes and compare their diagnostic performance with UTE-T2* mapping. We hypothesized that UTE-MT parameters would correlate well with the severity of knee OA, thereby providing a promising diagnostic and prognostic tool for detecting OA-related changes.

## 2. Materials and Methods

### 2.1. Subjects

This study was approved by the Institutional Review Board (IRB), and informed consent was obtained from all participants. Inclusion criteria included adults aged 19 to 90 years with a clinical OA diagnosis. Exclusion criteria included a history of knee surgery or trauma, knee tumors, or infectious lesions.

### 2.2. MRI Protocol

All participants were scanned using a 3T GE MRI scanner (MR750, GE Healthcare, Milwaukee, WI, USA) equipped with a transmit/receive 8-channel knee coil. Imaging was performed in the sagittal plane and included the following sequences: quantitative 3D UTE-MT for measuring MMF and MTR [28] and multi-echo UTE for T2* mapping. In addition, two-pool MT modeling for MMF mapping requires a measured T1 map as input, and T1 mapping was measured by a UTE actual flip angle and variable flip angle (VFA) technique [28,29]. A cones K-space trajectory was employed for UTE acquisitions [30]. Conventional clinical sequences included fat-suppressed T2-weighted (T2w) FSE and proton-density-weighted (PDw) FSE scans.

The detailed acquisition parameters for the quantitative 3D UTE imaging protocols and conventional clinical MRI sequences were as follows: (A) 3D UTE-MT sequence for MMF and MTR mapping: MT pulse flip angles (FAs)= 500°, 1000°, and 1500°; MT pulse frequency offsets = 2, 5, 10, 20, and 50 kHz; repetition time (TR) = 102 ms; echo time (TE) = 0.032 ms; excitation FA = 7°; number of spokes = 11; spoke interval (τ) = 6 ms; field of view (FOV) = 15 × 15 × 10.8 cm^3^; matrix size = 256 × 256 × 36; slice thickness = 3.0 mm; bandwidth = 166 kHz; (B) 3D fat suppressed multi-echo UTE sequence: TEs = 0.032, 4.4, 8.8, 13.2, 17.6, and 22 ms; TR = 33 ms, FA = 10°; (C) 3D UTE-VFA sequence: FAs = 5°, 10°, 20°, and 30°; TR = 20 ms; (D) 2D sagittal fat-suppressed T2-weighted FSE: FOV = 15 × 15 cm^2^; matrix size = 352 × 256; receiver bandwidth = 162 kHz; slice thickness = 3.0 mm; TR = 4713 ms; TE = 70 ms; (E) 2D sagittal proton-density-weighted FSE: TE = 27.6 ms; TR = 2128 ms; slice thickness and spacing = 3.0 mm; FOV = 15 × 15 cm^2^; matrix size = 352 × 256. The total scan time was approximately 26 min. The following UTE-MT dataset was utilized for MTR mapping: MT pulse FA = 1500° (MT on) and 0° (MT off) and frequency offset = 2 kHz.

### 2.3. Data Analysis

Elastix-based motion registration was applied to each patient’s dataset [31]. Initially, a rigid affine transformation was used for coarse alignment between images, followed by a nonrigid B-spline registration to achieve more precise spatial alignment.

A board-certified radiologist, blinded to the UTE quantitative data, assessed the patients’ knee osteoarthritis using the clinical Kellgren–Lawrence (KL) grading system [32] on anteroposterior (AP) plain radiographs. Patients were classified into four groups: normal (KL score 0), subtle (KL score 1), mild (KL score 2), and moderate (KL score 3) OA.

**Figure 1:**
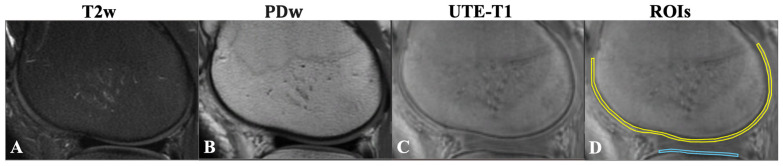
(**A**) Fat-suppressed T2w-FSE (T2w). (**B**) Proton-density-weighted FSE (PDw). (**C**) UTE-T1-weighted (FA = 30°) images of the knee joint for a 38-year-old male with KL = 0. Note that the femoral condyle OCJ (yellow line) and tibial condyle OCJ (blue line) show no signal on conventional clinical MRI images (**A**,**B**). On the UTE-T1 image, the OCJ is evident as a region with a higher signal between the subchondral bone and deep cartilage (**C**). Using the UTE-T1 image, schematic regions of interest were placed in the femoral condyle OCJ (yellow line) and tibial condyle OCJ (blue line) for measurement as shown (**D**). T2w, T2-weighted; PDw, proton-density-weighted; OCJ, osteochondral junction; UTE-T1, ultrashort echo time-based T1; KL, Kallgren–Lawrence.

Quantitative UTE MRI data analysis was performed by an experienced postdoctoral scholar, blinded to the patient group and clinical data, who performed the image analysis using MATLAB (2023, The MathWorks Inc., Natick, MA, USA) on the registered images. Within each slice, four distinct regions of interest (ROIs) were selected at the OCJ: one on the femoral condyle, manually subdivided into anterior (AFC), inferior (IFC), and posterior (PFC) segments, and the other on the tibial plateau (TP). The OCJ was defined as the area between the edge of the subchondral bone and the adjacent deep cartilage (Figure 1D). Considering the knee as an entire organ, and given that the KL scoring system does not differentiate cartilage segments, the average UTE measurements of the four different cartilage regions across the selected slices in each knee were calculated and used in the final analysis for each patient. Two-pool MT modeling, which characterizes the interaction between water and macromolecular protons, was employed to derive MMF values [28]. UTE-MTR was calculated using two UTE datasets acquired with saturation powers of 1500° (MT-on) and 0° (MT-off), respectively. Single-component fitting models acquired from the multi-echo 3D-UTE-Cones sequence were utilized for T2* decay analyses.

### 2.4. Statistical Analysis

The Kolmogorov–Smirnov test was used to assess the normality of the MMF, MTR, and T2* distributions. Since the MMF and MTR values were not normally distributed, the Mann–Whitney test was employed to compare MTR values across different OA grade groups. In contrast, T2* values, which followed a normal distribution, were compared using an independent T-test. The patients were categorized into two groups based on OA severity: normal/subtle (KL = 0 and 1) and mild to moderate (KL = 2 and 3).

Spearman’s correlation coefficient was used to assess the relationship between MMF and MTR values and the clinical KL classification, as these variables did not meet the normality assumption. Pearson’s correlation coefficient was used to evaluate the correlation between T2* values and the KL classification, as T2* was normally distributed. Receiver operating characteristic (ROC) curve analyses were performed to calculate the area under the curve (AUC) for MMF, MTR, and T2* in distinguishing osteoarthritis severity. This analysis categorized OA grade groups as normal/subtle (KL = 0 and 1) and mild to moderate (KL = 2 and 3).

To assess inter-reader reliability and validity, intraclass correlation coefficients (ICCs) were calculated between the results from two independent analysts. Intra-reader reliability was evaluated by calculating ICCs comparing two repeated measurements performed on ten randomly selected participants by the first data analyst.

A *p*-value of less than 0.05 was considered statistically significant. All statistical analyses were performed using SPSS Statistics version 29.0 (IBM Corp., Armonk, NY, USA).

## 3. Results

A total of sixty subjects participated in this study (29 females and 31 males), with an age range of 20 to 88 years (mean ± SD: 53.6 ± 17.1 years). However, due to severe motion artifacts that could not be corrected retrospectively and the exclusion of cases with advanced osteoarthritis (KL = 4), where deep cartilage was extensively degraded, the final analysis included 45 patients with OA (mean age: 53.8 ± 17.0 years; 50% female).

The number of participants of different Kellgren–Lawrence grades was as follows: KL score 0: 17; KL score 1: 4; KL score 2: 9; and KL score 3: 15.

The mean ± standard deviation (SD) values of the UTE-MRI biomarkers (MMF, MTR, and T2*) across the OA groups are summarized in Table 1. As the number of patients in each group is relatively small, the median and interquartile range (IQR) values for MRI biomarkers are presented in Supplementary Table S1.

**Figure 2:**
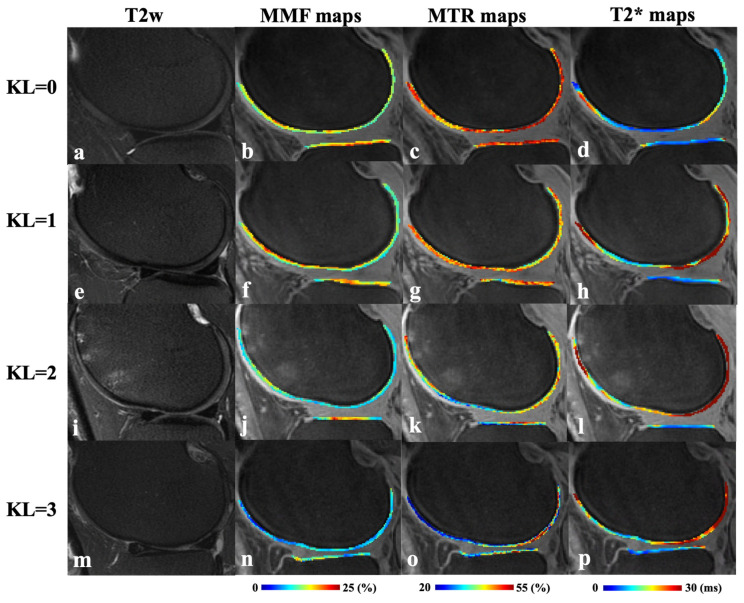
Representative fat-suppressed T2w-FSE (T2w) images (**a**,**e**,**i**,**m**) and corresponding MMF (**b**,**f**,**j**,**n**), MTR (**c**,**g**,**k**,**o**), and T2* (**d**,**h**,**l**,**p**) maps from four participants with varying degrees of KL scores. Top row (**a**–**d**): a 23-year-old male with normal cartilage (KL = 0); second row (**e**–**h**): a 57-year-old male with subtle cartilage changes (KL = 1); third row (**i**–**l**): a 52-year-old female with mild cartilage degeneration (KL = 2); bottom row (**m**–**p**): a 70-year-old female with moderate cartilage degeneration (KL = 3). T2w, T2-weighted fast spin echo; MMF, macromolecular fraction; MTR, magnetization transfer ratio.

Figure 2 presents representative fat-suppressed T2w-FSE images and quantitative maps of MMF, MTR, and T2* from four participants with varying degrees of OA, classified by KL scores. In the healthy subject with KL = 0 (23-year-old male), the MMF was 15.4 ± 2.2%, MTR was 46.7 ± 5.8%, and T2* was 18.1 ± 6.1 ms. In the subject with KL = 1 (57-year-old male), subtle cartilage changes were associated with a slightly higher MMF of 15.8 ± 3.8% but reduced MTR of 40.6 ± 4.9% and T2* of 18.7 ± 6.1 ms. The slightly higher MMF value in KL 1 can be explained that the degeneration in this KL 1 subject is subtle. For the participant with KL = 2 (52-year-old female), MMF declined to 13.2 ± 1.8% and MTR to 36.2 ± 5.1%, while T2* remained relatively stable at 19.0 ± 11.1 ms. In the participant with more advanced cartilage degeneration (KL = 3, 70-year-old female), MMF further decreased to 11.1 ± 3.0%, MTR to 30.8 ± 1.1%, while T2* increased to 22.8 ± 7.3 ms. These results demonstrate a general trend of decreasing MMF and MTR with advancing OA, while T2* showed more variability and less consistent correlation with disease severity.

Excellent inter-reader reliability was observed for all the UTE measurements: MMF: 0.94; MTR: 0.92; and T2*: 0.90. Also, excellent intra-reader reliability was observed for all the UTE measurements: MMF: 0.96; MTR: 0.94; and T2*: 0.92 [33].

**Figure 3:**
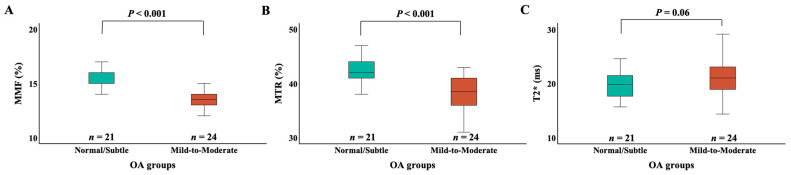
Box plots of the MMF (**A**), MTR (**B**), and T2* (**C**) measurements for different OA groups. The “Normal/Subtle” OA group includes KL grades 0 and 1, and the “Mild to Moderate” OA group includes KL grades 2 and 3. The edges of the box indicate the first and third IQR percentiles, respectively. The normal/subtle OA group had significantly higher MMF than the mild to moderate OA group (*p* < 0.001). The normal/subtle OA group had significantly higher MTR than the mild to moderate OA group (*p* < 0.001). The normal/subtle OA group had lower T2* than the mild to moderate OA group (*p* = 0.06). KL, Kallgren–Lawrence; OA, osteoarthritis; MMF, macromolecular fraction; MTR, magnetization transfer ratio; IQR, interquartile range.

Figure 3 presents box plots of MMF, MTR, and T2* values across different OA groups. Patients with the normal/subtle OA group demonstrated significantly higher MMF (15.8 ± 1.4% vs. 13.6 ± 1.2%, *p* < 0.001) and MTR (42.5 ± 2.5% vs. 38.3 ± 2.9%, *p* < 0.001) values compared to those with the mild to moderate OA group. The normal/subtle OA group had lower T2* than the mild to moderate OA group but did not reach statistical significance (19.7 ± 2.6 ms vs. 21.6 ± 3.8 ms, *p* = 0.06).

**Figure 4:**
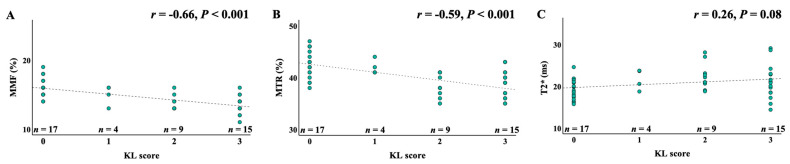
Pearson’s and Spearman’s correlations between MMF, MTR, and T2* measurements and the KL scores. (**A**) Spearman’s correlation between MMF values and KL scores; (**B**) Spearman’s correlation between MTR values and KL scores; (**C**) Pearson’s correlation between UTE-T2* values and KL scores. The KL scores are negatively correlated with both MMF (*r* = −0.66, *p* < 0.001) and MTR (*r* = −0.59, *p* < 0.001) and weakly positively correlated with T2* (r = 0.26, *p* = 0.08), KL, Kallgren–Lawrence; MMF, macromolecular fraction; MTR, magnetization transfer ratio.

A correlation analysis between UTE-MRI biomarkers and KL scores is shown in Figure 4. MMF and MTR demonstrated significant negative correlations with KL scores (*r* = −0.66 and −0.59, respectively; *p* < 0.001), indicating that macromolecular content decreases with increasing OA severity. T2* values showed a weaker but statistically significant positive correlation (*r* = 0.26, *p* = 0.08), reflecting increased water content and disorganization in more advanced OA.

**Figure 5:**
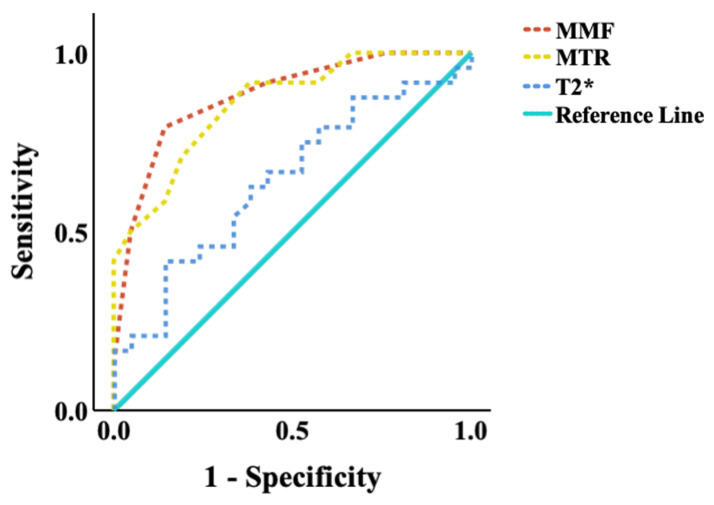
ROC curves of quantitative UTE-MRI measurements for distinguishing between subjects with normal/subtle OA findings (KL scores 0 and 1) and those with significant OA findings (KL scores 2 and 3). The areas under the curve (AUC) of MMF (AUC  =  0.88, *p* < 0.001), followed by MTR (AUC  =  0.86, *p* < 0.001), were much higher than T2* (AUC  =  0.64, *p* = 0.08) at a 0.05 significance level. UTE, ultrashort echo time; MMF, macromolecular fraction; MTR, magnetization transfer ratio.

The diagnostic performance of the UTE biomarkers was assessed using ROC analysis (Figure 5). MMF achieved the highest area under the curve (AUC = 0.88, *p* < 0.001), followed by MTR (AUC = 0.86, *p* < 0.001). T2* mapping showed limited diagnostic value in distinguishing low-grade OA stages (AUC = 0.64, *p* = 0.08). These results support the superior performance of MMF and MTR as early imaging biomarkers for detecting biochemical changes in the OCJ region associated with OA progression. The optimal diagnostic threshold identified for MMF was 15%, providing a sensitivity of 79.2% and specificity of 82.6%. For MTR, the optimal threshold was 41.5%, resulting in a sensitivity of 92.3% and a specificity of 60.9%. The optimal cut-offs for MMF and MTR are 40.5% and 15.0%, respectively, based on Youden’s Index maximization.

## 4. Discussion

This study is the first to assess the OCJ region of knee cartilage using whole-knee 3D MMF, MTR, and T2* imaging on a clinical 3T MRI scanner in patients with varying stages of OA. Significant moderate correlations were observed between UTE-MT measurements (i.e., MMF and MTR) in the OCJ and corresponding KL grades, while T2* measurements showed a weak correlation with KL grades. ROC analysis demonstrated that MMF and MTR values were reduced in patients with low-grade OA compared to those with more advanced diseases. Among the evaluated imaging biomarkers, MMF exhibited the highest diagnostic sensitivity and specificity, followed closely by MTR, whereas T2* showed lower discriminatory performance. These results support the potential of MMF and MTR as sensitive and quantitative imaging biomarkers for evaluating OCJ degeneration in OA.

The OCJ is a metabolically active interface between the articular cartilage and subchondral bone [27,32,34]. It is characterized by a distinct composition that includes type X collagen [35] and exhibits an extremely thin structural profile [36]. In the deep layers of knee cartilage, the proteoglycan (macromolecular) content increases progressively with depth from the articular surface [37]. Moreover, collagen fibrils in this region become thicker and are oriented more perpendicularly to the subchondral bone surface [38], reflecting the functional adaptation of the tissue to mechanical loading.

The OCJ plays a critical role in the pathogenesis of knee OA [39]. However, imaging the deeper regions of knee cartilage, including the OCJ, using conventional noninvasive MRI techniques remains challenging due to the tissue’s intrinsic characteristics—namely, low water content and high collagen concentration—which result in extremely short T2 and T2* relaxation times [40]. In recent years, the development of UTE-MRI has enabled both qualitative and quantitative assessment of these deep cartilage layers in vivo [25,33], offering new potential for the early detection and evaluation of OA.

Quantitative UTE imaging techniques, including UTE-MT and UTE-T2* mapping, have been investigated for their potential in evaluating cartilage degeneration [22,24,25,41,42,43,44,45,46,47,48,49]. Among these techniques, two-pool UTE-MT modeling is particularly noteworthy, as it enables the quantification of MMF and MTR. These parameters provide direct insight into the macromolecular proton pool and its relationship to free water, offering unique information about cartilage composition and degeneration [27,28,50]. Notably, UTE-MT modeling is relatively insensitive to the magic angle effect [27,48,51], a common source of concern in T2 or T2* measurements, especially in the deep layers of the knee joint.

In a prospective study by Xue et al., a two-pool MT model was applied to assess MMF for differentiating normal and degenerated knee cartilage [26]. Analyzing data from 62 volunteers with and without OA, the authors found that MMF values in full-thickness cartilage were negatively correlated with both KL grades and Whole-Organ Magnetic Resonance Imaging Scores (WORMSs), highlighting MMF’s potential as a biomarker for early OA detection. Our findings are consistent with these results. In another study, Yang et al. evaluated 20 degenerative anterolateral condyles obtained from total knee arthroplasty specimens and compared UTE-MTR values with those from T2* and T2 mapping [22]. Their results demonstrated strong correlations between MTR and Mankin histological scores—an established marker of cartilage degeneration—further showing that UTE-MTR had superior diagnostic performance in detecting early cartilage degeneration compared to UTE-T2* and T2 mapping. Similarly, Shao et al. reported that UTE-MTR exhibited the strongest correlation with the Osteoarthritis Research Society International (OARSI) grade and the polarized light microscopy (PLM) collagen organization score [42]. UTE-MTR could distinguish between normal and mildly degenerated cartilage, whereas UTE-T2* and CubeQuant-T2 could not. Their study identified UTE-MTR as a highly effective diagnostic biomarker for early cartilage degeneration.

UTE-T2* mapping has been widely utilized in previous studies and has shown promise in detecting early degenerative changes in cartilage. Williams et al. reported that UTE-T2* mapping is sensitive to matrix alterations, demonstrating lower T2* values in regions with higher matrix degeneration based on the David-Vaudey grading scale [44]. In a separate study, the same group showed that UTE-T2* imaging could distinguish early cartilage lesions (Outerbridge grades 0 and 1) from normal cartilage in a cohort of patients with anterior cruciate ligament (ACL) injuries [46]. Additionally, they demonstrated that UTE-T2* imaging could detect changes in the calcified cartilage layer in postoperative knees, correlating with clinical markers indicative of an increased risk for medial knee OA [47]. Despite these findings, the UTE-T2* assessment remains challenging due to its sensitivity to several confounding factors, particularly the magic angle effect [27,32], which may have influenced our results and contributed to the relatively weak correlation observed between T2* values and KL scores. Furthermore, variations in UTE imaging protocols, cartilage degeneration scoring methods, and differences between in vivo and ex vivo study designs may also account for discrepancies between our findings and those of previous studies.

Unlike conventional MRI techniques that primarily assess structural changes or fluid content, our study suggests that both quantitative MMF and MTR biomarkers are promising in detecting macromolecular degradation associated with early disease progression. This degradation primarily results from the breakdown of proteoglycans and collagen in the extracellular matrix. Compared to T2* mapping, which is influenced by factors such as collagen fiber orientation and water content, MTR offers a more specific indication of macromolecular changes but remains semi-quantitative and lacks direct measurement of macromolecular and water proton content [28]. In contrast, MMF provides a more direct and quantitative assessment of macromolecular content, making it a potentially more sensitive and specific biomarker for identifying early cartilage degeneration [22]. The quantitative UTE-MT technique could serve as a more precise diagnostic tool for identifying early-stage OA, allowing for timely interventions before irreversible cartilage loss occurs. Furthermore, these quantitative biomarkers offer a potential avenue for more effective disease monitoring, enabling clinicians to track therapeutic efficacy or disease progression objectively, thereby facilitating personalized treatment strategies in the management of knee OA.

This study has several limitations. First, the sample size was relatively small, and larger cohorts are needed to confirm and generalize the findings. Second, the clinical implementation of UTE-MT is currently constrained by prolonged acquisition times, technical demands, and post-processing complexity. However, the integration of artificial intelligence and machine learning could streamline various aspects of UTE-MT, including scan acceleration, automated reconstruction, and intelligent segmentation. We believe these advancements will be key to enhancing the clinical feasibility and widespread adoption of this promising technique. Third, future work should investigate the relationship between MMF and histopathological changes in articular cartilage.

## 5. Conclusions

This study demonstrated that MMF and MTR values were significantly correlated with OA severity, and they outperformed T2* mapping in detecting OA changes. The UTE-MT technique is promising to serve as a clinical evaluation tool for the comprehensive noninvasive diagnosis and longitudinal monitoring of OA.

## Figures and Tables

**Table 1:** Mean and standard deviation (SD) values of estimated ultrashort echo time magnetic resonance imaging (UTE-MRI) biomarkers (MMF, MTR, and T2*) in different OA groups. MMF, macromolecular fraction; MTR, magnetization transfer ratio.

Patient Groups	MMF (%)	MTR (%)	T2* (ms)
**Normal/Subtle (*n* = 21)**	15.8 ± 1.4	42.5 ± 2.5	19.7 ± 2.6
**Mild to Moderate (*n* = 24)**	13.6 ± 1.2	38.3 ± 2.9	21.6 ± 3.8

## Data Availability

The data presented in this study are available on request from the corresponding author. The data are not publicly available due to patient confidentiality.

## Supplementary Materials

The following supporting information can be downloaded at: https://www.mdpi.com/article/10.3390/jimaging11060198/s1, Table S1: Median and Interquartile Range (IQR) of UTE-MRI biomarkers measurements (MMF, MTR, T2*) in different OA groups.

## Abbreviations

OCJ, osteochondral junction; OA, osteoarthritis; MRI, magnetic resonance imaging; UTE, ultrashort echo time; 3D, three-dimensional; MTR, magnetization transfer ratio; MMF, macromolecular fraction; RF, radiofrequency; FOV, field of view; ROI, region of interest; TE, echo time; TR, repetition time; FA, flip angle; FSE, fast spin echo; GRE, gradient echo; IR, inversion recovery; DIR, dual inversion recovery; CNR, contrast-to-noise ratio; MT, magnetization transfer; VFA, variable flip angle; KL, Kellgren–Lawrence; ANOVA, analysis of variance; ROC, receiver operating characteristic; AUC, area under the curve; ICC, intraclass correlation coefficient; SD, standard deviation; FS, fat-suppressed; PD, proton-density; AP, anteroposterior; WORMS, whole-organ magnetic resonance imaging score; PLM, polarized light microscopy; OARSI, Osteoarthritis Research Society International; ACL, anterior cruciate ligament
