# Supplementary material for: Quantitative Ultrashort Echo Time Magnetization Transfer Imaging of the Osteochondral Junction: An In Vivo Knee Osteoarthritis Study
Source: J Imaging. 2025 Jun 16;11(6):198. doi: 10.3390/jimaging11060198 (PMC12193826; doi:10.3390/jimaging11060198)
Supplement: Supplementary file 1 [file jimaging-11-00198-s001.zip › jimaging-3659018-supplementary.pdf]

Table S1. Median and Interquartile Range (IQR) of UTE-MRI biomarkers measurements (MMF, MTR, T2*) in different OA groups

**KL scores**	**MMF (%)**	**MTR (%)**	**T2* (ms)**
**Normal/Subtle (*n* = 21)**	16.0 [1.5]	42.7 [3.5]	19.8 [4.1]
**Mild to Moderate (*n* = 24)**	13.5 [1.0]	38.5 [5.0]	21.8 [4.2]
